# Comparative Pollen Morphological Analysis and Its Systematic Implications on Three European Oak (*Quercus* L., Fagaceae) Species and Their Spontaneous Hybrids

**DOI:** 10.1371/journal.pone.0161762

**Published:** 2016-08-26

**Authors:** Dorota Wrońska-Pilarek, Władysław Danielewicz, Jan Bocianowski, Tomasz Maliński, Magdalena Janyszek

**Affiliations:** 1 Department of Forest Botany, Poznan University of Life Sciences, Poznań, Poland; 2 Department Mathematical and Statistical Methods, Poznan University of Life Sciences, Poznań, Poland; 3 Department of Botany, Poznan University of Life Sciences, Poznań, Poland; Aristotle University of Thessaloniki, GREECE

## Abstract

Pollen morphology of three parental *Quercus* species (*Q*. *robur* L., *Q*. *petraea* (Matt) Liebl, *Q*. *pubescens* Willd.) and two spontaneous hybrids of these species (*Q*. ×*calvescens* Vuk. = *Q*. *petraea* × *Q*. *pubescens* and *Q*. *×rosacea* Bechst. *= Q*. *robur × Q*. *petraea*) was investigated in this study. The pollen originated from 18 natural oak sites and 67 individuals (oak trees). Each individual was represented by 30 pollen grains. In total, 2010 pollen grains were measured. They were analysed for nine quantitative and four qualitative features. Pollen size and shape were important features to diagnosing *Quercus* parental species and hybrids. On the basis of exine ornamentation, it was possible to identify only *Q*. *pubescens*, while the remaining species and hybrids did not differ significantly with respect to this feature. The determination of the diagnostic value of endoaperture features requires further palynological studies. On the basis of pollen size and shape *Q*. *robur* × *Q*. *petraea* was clearly separated. Grouping of 67 oak trees on the basis of pollen grain features has shown that individuals from different as well as same taxa occurred in the same groups. Likewise, with respect to natural sites, oak trees originating from the same places as well as from geographically distant ones, grouped together. Pollen morphological features allow to distinguish a part of the studied *Quercus* taxa. Therefore, it can be used as an auxiliary feature in the taxonomy.

## Introduction

*Quercus* genus is the largest genus in the Fagaceae family. Depending on different authorities, the number of oak species varies between 300 and 400 [1–5], through 500–531 [6, 7] up to 600 [8, 9]. The members of this genus occupy territories of the Northern Hemisphere in Asia, North America, Europe and Africa, with a few species extending to the equator [10–14].

The taxonomy of *Quercus* genus is extremely complex as a result of high species numbers, wide geographical distribution, great morphological variability, as well as widespread hybridization between infrageneric taxa and changes in morphological features [7, 8, 15–21]. Therefore, the classification of oaks has been a matter of debate, from De Candolle [22] to Nixon [13], and more than 20 classifications were proposed [16, 23].

Up to date, the most taxonomically valuable morphological features of *Quercus* species are foliar and fruit characteristics. Hence, the taxonomic classifications of oaks are usually based on these features [5, 13, 15, 24, 25]. According to Menitski’s [11] classification, the three species analyzed for the present study belong to the *Quercus* L., subgenus *Quercus*, section *Quercus*, series *Quercus* and the subseries: *Quercus*—*Quercus robur* L., *Quercus petraea* (Matt.) Liebl. and *Galliferae* (Spach) Guerke—*Quercus pubescens* Willd. According to the infrageneric groups recognized by Denk and Grimm [26] the three species belong to the *Quercus*, Group *Quercus*.

Occurrence and frequency of interspecific hybrids in natural European oak populations has not been clarified satisfactorily so far [27]. In the botanical literature, interspecific hybrids in natural European oak populations have been distinguished for a long time, primarily on the basis of morphological traits of indirect characters between the alleged parental species [1, 24, 28]. They comprise, among others, such taxons as: *Quercus* ×*calvescens* Vuk. (*Q*. *petraea* × *Q*. *pubescens*), *Q*. ×*kerneri* Simk. (*Q*. *robur* × *Q*. *pubescens*) and *Q*. ×*rosacea* Bechst. (*Q*. *robur* × *Q*. *petraea*).

According to Rushton [20], in mixed populations of *Q*. *robur* and *Q*. *petraea*, the proportion of trees morphologically “truly intermediate” between the above species in north-western Europe, estimated on the basis of data quoted by different researchers, ranges from 2 to 29%. Many authors think that hybrids between these species are rare, and frequency of their appearance in natural population is approximately 2–3% [15, 29–35].

However, detailed comparative, palynological studies of pollen grain characters between oak hybrids and parental species have not been performed. These characters were described for taxonomic purposes in numerous studies using scanning electron microscopy (SEM) [17, 36–48]. Many researchers maintain, that pollen morphology can be a valuable source of information for *Quercus* taxonomy [17, 36–40, 43, 45, 46], whereas others contest the possibility of differentiating species through characteristics of pollen grains, since intraspecific variability may appear to be the same or greater than interspecific differences [49–52].

Palynological studies on interspecific hybrids concern artificial hybrids [53–59], or spontaneous hybrids found in nature [60–65]. In these studies only a few, selected quantitative pollen features are usually compared; most commonly they include pollen size [55, 57–60, 62–66], more rarely—exine ornamentation [38, 40, 53, 54, 56] or aperture numbers [54, 55]. Only few researchers investigated also the different degrees of deformed pollen grains deformation in parental species and hybrids [60, 64].

A recent pollen morphological study focusing on pollen ornamentation [38] showed that this pollen character is diagnostic for six major infrageneric groups within *Quercus*. These six infrageneric groups are also recognized by molecular phylogenetic studies [26, 67, 68].

Therefore, the aim of our study was to evaluate whether and how pollen grains of the three studied parental species (*Q*. *robur* L., *Q*. *petraea* (Matt) Liebl., *Q*. *pubescens* Willd.) differ from those of their spontaneous hybrids: *Quercus* ×*calvescens* Vuk. (*Q*. *petraea* × *Q*. *pubescens*), and *Q*. × *rosacea* Bechst. (*Q*. *robur* × *Q*. *petraea*). This has not been the object of palynological studies so far. In addition, pollen grain variability of the investigated oak taxa has not yet been comprehensively analysed. We think that one of the strengths of this study is that we take advantage of a relatively large sample from several individuals, in order to capture much of intraspecific variability, in contrast to other studies, that focus in distinguishing different phylogenetic groups [44–46, 49].

## Material and Methods

While gathering sufficiently large samples from typical *Q*. *robur*, *Q*. *petraea* and *Q*. *pubescens* individuals was not difficult, the collection of inflorescences from trees morphologically intermediate between them (assumed hybrids) was considerably limited, because of their rare occurrence. The assumed hybrids collected from Bielinek, the sole *Q*. *pubescens* site in Poland (52°56'26"N, 14°8'54"E) comprised mainly *Q*. *petraea × Q*. *pubescens* hybrids, although single specimens of *Q*. *robur × Q*. *pubescens* cannot be excluded. Plant materials in the form of fresh inflorescences was selected and verified by Professor Władysław Danielewicz (Department of Forest Botany, Poznań University of Life Sciences), whereas, that from the Herbarium of the Institute of the Dendrology of Polish Academy of Sciences in Kórnik was verified by Professor Jerzy Zieliński. In this study, pollen morphology of three parental *Quercus* species (*Q*. *robur* L., *Q*. *petraea* (Matt) Liebl, *Q*. *pubescens* Willd.) and two spontaneous hybrids of these species (*Quercus* ×*calvescens* Vuk. = *Q*. *petraea* × *Q*. *pubescens* and *Q*. *×rosacea* Bechst. *= Q*. *robur × Q*. *petraea*) were analysed (Table 1).

**Table 1:** List of localities of the studied *Quercus* taxa.

No	Species-Taxon	Localities	Coordinates	Collector, herbarium	No of trees (samples) for each location
1	*Q*. *petraea* (Matt) Liebl.	Dębno, zachodniopomorskie, Poland	52°44'20"N, 14°41'52"E	Danielewicz; PZNF	5
2	*Q*. *petraea* (Matt) Liebl.	Poznań - Marcelin, wielkopolskie, Poland	52°24'23"N, 16°51'86"E	Danielewicz; PZNF	2
3	*Q*. *petraea* (Matt) Liebl.	Puszcza Bukowa, zachodniopomorskie, Poland	53°20'12"N, 14°40'29"E	Danielewicz; PZNF	5
4	*Q*. *petraea* (Matt) Liebl.	Rokita, zachodniopomorskie, Poland	53°45'53"N, 14°50'27"E	Danielewicz; PZNF	3
5	*Q*. *petraea* (Matt) Liebl.	Różańsko, zachodniopomorskie, Poland	52°50'56"N, 14°46'54"E	Danielewicz; PZNF	5
6	*Q*. *petraea* (Matt) Liebl.	Wielkopolski National Park, wielkopolskie, Poland Province	52°16'94"N, 16°47'57"E	Danielewicz; PZNF	5
7	*Q*. *petraea* (Matt) Liebl.	Vitosha Mts., Bulgaria, 950 m	42°34'00"N, 23°17'00"E	Vihodcevsky; KOR	1
8	*Q*. *petraea* (Matt) Liebl.	Vall de Ribes, Pyrenees Mts., Spain, 1500 m	42°37′56"N, 0°39′28"E	Boratyński; KOR	1
9	*Q*. *pubescens* Willd.	Bielinek, zachodniopomorskie, Poland	52°56′26"N, 14°8'54"E	Maliński; PZNF	10
10	*Q*. *pubescens* Willd.	Hafnerberg, Austria	48°01′00"N, 16°01′00"E	Browicz; KOR	1
11	*Q*. *pubescens* Willd.	Perithia, Korfu, Greece	39°76′46"N, 19°87′54"E	Boratyński, Browicz; KOR	1
12	*Q*. *pubescens* Willd.	Crimea, Russia	45°18′00"N, 34°24′00"E	Dzewanowskaya; KOR	1
13	*Q*. *robur* L.	Białowieża, podalskie, Poland	52°45′76″N 23°52′45″E	Danielewicz; PZNF	5
14	*Q*. *robur* L.	Jarocin, wielkopolskie, Poland	51°58'23"N, 17°30'12"E	Danielewicz; PZNF	2
15	*Q*. *robur* L.	Promno, wielkopolskie, Poland	52°27'37"N, 17°14'44"E	Danielewicz; PZNF	5
16	*Q*. *robur* L.	Puszcza Bukowa, zachodniopomorskie, Poland	53°20'12"N, 14°40'29"E	Danielewicz; PZNF	1
17	*Q*. *robur* L.	Rogalin, wielkopolskie, Poland	52°14'43"N, 16°56'27"E	Danielewicz; PZNF	2
18	*Q*. *robur* L.	Rokita, zachodniopomorskie, Poland	53°45'53"N, 14°50'27"E	Danielewicz; PZNF	5
19	*Q*. *robur* L.	Wielkopolski National Park, wielkopolskie, Poland	52°16'94"N, 16°47'57"E	Danielewicz; PZNF	2
20	*Q*. *robur* L	Andrusul de Sus, Moldova	46°0′20"N, 28°14′51"E	Poneasheskij; KOR	1
21	*Q*. ×*calvescens* Vuk. = *Q*. *petraea × Q*. *pubescens*	Bielinek, zachodniopomorskie, Poland	52°56'26"N, 14°8'54"E	Maliński; PZNF	3
22	*Q*. *×rosacea* Bechst. *= Q*. *robur × Q*. *petraea*	Poznań, Dendrological Garden of Poznan University of Life Sciences, wielkopolskie, Poland	52°25'40"N, 16°53'57"E	Danielewicz; PZNF	1

KOR—Herbarium of the Institute of Dendrology, Polish Academy of Sciences, Kórnik, Poland; PZNF—Herbarium of Department of Forest Botany, Poznan University of Life Sciences, Poland.

Male inflorescences investigated for this study originate from 18 natural oaks sites, located in Austria, Bulgaria, Greece (Corfu), Spain, Crimea, Moldova and Poland. Except for the Polish material, male inflorescences were obtained from herbarium material stored in the Herbarium of the Institute of Dendrology of the Polish Academy of Sciences in Kórnik (52°14'12''N, 17°05'55''E)–KOR (Poland) (Table 1). Several, randomly selected inflorescences were collected from each of 67 oak individuals. Each individual is represented by 30 correctly formed, mature pollen grains [69]. In total, 2010 pollen grains were measured. Malformed pollen grains were also noticed in the samples, and their percentage was determined considering 1000 pollen grains in *Q*. *petraea* and *Q*. *robur* (five randomly selected samples of 200 pollen grains), and 600 grains in *Q*. *pubescens* and *Q*. *petraea × Q*. *pubescens* (three samples) and 200 pollen grains in the rare hybrid *Q*. *robur × Q*. *petraea*.

For the measurements, samples were acetolysed according to Erdtman’s method [70]. The acetolysing mixture was made up of nine parts of acetic acid anhydride and one part of concentrated sulphuric acid and the process of acetolysis lasted 2.5 minutes. The measurements were made on acetolysed grains with light microscope (Biolar 2308) and observations of qualitative features were carried out with scanning electron microscope (Hitachi S-3000N) on acetolysed grains to.

Pollen grains were prepared in glycerine jelly and measured using the eyepiece (ocular) with scale. Next, the pollen grains were analysed for nine quantitative features, i.e. length of polar axis (P), equatorial diameter (E), length of ectocolpi (Le), thickness of exine along polar axis (Exp) and equatorial diameter (Exe) and four ratios: P/E, Exp/P, Exe/E, Le/P; and the following qualitative ones: exine ornamentation, endoaperture type, pollen outline and shape.

The palynological terminology follows Punt et al. [71] and Hesse et al. [72].

Firstly, the normality of the distributions of the studied traits (P, E, P/E, Exp, Exe, Exp/P, Exe/E, Le and Le/P) was tested using the Shapiro-Wilk’s normality test [73]. Multivariate analysis of variance (MANOVA) was performed on the basis of the following model using a procedure MANOVA in GenStat 17th edition: **Y** = **XT**+**E**, where: **Y** is (*n*×*p*)–dimensional matrix of observations, *n* is number of all observations, *p* is number of traits (in this study *p* = 9), **X** is (*n*×*k*)–dimensional matrix of design, *k* is number of taxa (in this study *k* = 5), **T** is (*k*×*p*)—dimensional matrix of unknown effects,—is (*n*×*p*)–dimensional matrix of residuals. Next, one-way analyses of variance (ANOVA) were performed in order to verify the zero hypothesis on a lack of taxon effect in terms of values of observed traits, i.e. P, E, P/E, Exp, Exe, Exp/P, Exe/E, Le and Le/P for each trait independent, on the basis of the following model: *y*_*ij*_ = *μ*+*τ*_*i*_+*ε*_*ij*_, where: *y*_*ij*_ is *j*th observation of *i*th taxon, *μ* is grand mean, *τ*_*i*_ is effect of *i*th taxon and *ε*_*ij*_ is an error observation. The minimal and maximal values of traits as well as arithmetical means and coefficients of variation—CV (in %)—were calculated. Moreover, the Fisher’s least significant differences (LSDs) were also estimated at the significance level α = 0.001. The relationships between the observed traits were assessed on the basis of Pearson’s correlation coefficients using the FCORRELATION implementation in GenStat 17th edition. The parallel coordinate plot has been proposed as an efficient tool for visualization of species and their hybrids visualization [74, 75]. Results were also analysed using multivariate methods. The analysis of canonical variables was applied in order to present multitrait assessment of similarity of tested genotypes (two separate analyses: first for species and hybrids and second for trees) in a lower number of dimensions with the least possible loss of information [76]. This makes it possible to illustrate variation in genotypes in terms of all observed traits a graphic way. Mahalanobis’ distance was suggested as a measure of “polytrait” genotypes similarity [77], whose significance was verified by means of critical value D_α_ called “the least significant distance” [78]. Mahalanobis’ distances were calculated for taxa and trees, independently. All the analyses were conducted using the GenStat 17th edition statistical software package [79].

## Results

### General pollen morphology

**Fig 1:**
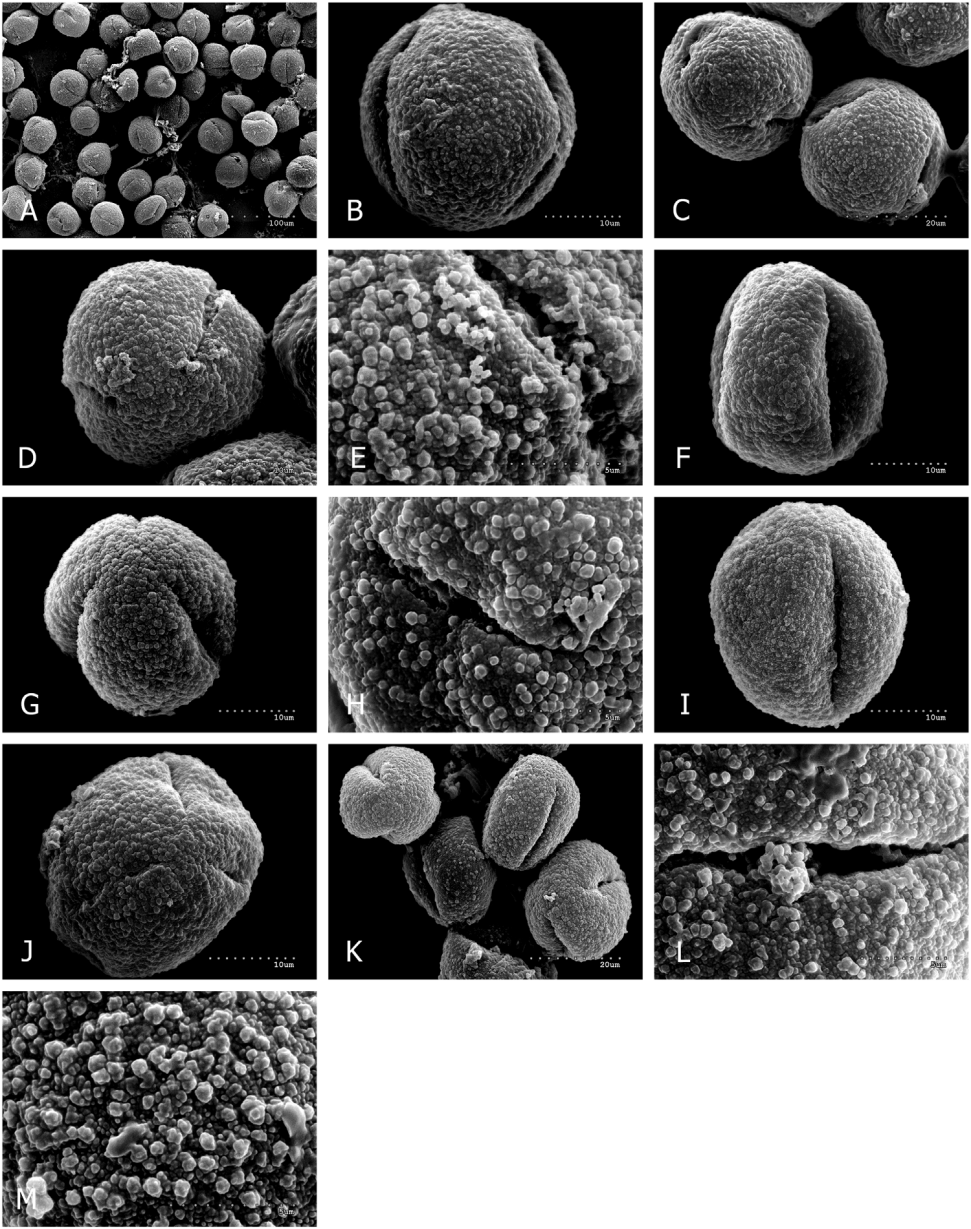
*Q*. *petraea*, A-E. A, numerous pollen grains in polar and equatorial view, spheroidal or prolate-spheroidal in shape; B, equatorial view; C, polar and equatorial view of two pollen grains; D, polar view with two ectocolpi; E, ectocolpus and granulate-verrucate exine ornamentation with the biggest verrucae (>1 μm), slightly smaller granules (0.5–1μm), and the smallest and low granules (0.1–0.3 μm); *Q*. *pubescens*, F-H. F, equatorial view; G, polar view with three ectocolpi; H, ectocolpus and granulate-verrucate exine ornamentation without small granules; *Q*. *robur*, I-M. I, equatorial view; J, polar view with three, closed ectocolpi; K, four pollen in polar and equatorial view; L, ectocolpus and granulate-verrucate exine ornamentation; M, exine ornamentation details—see (Fig 1E).

**Fig 2:**
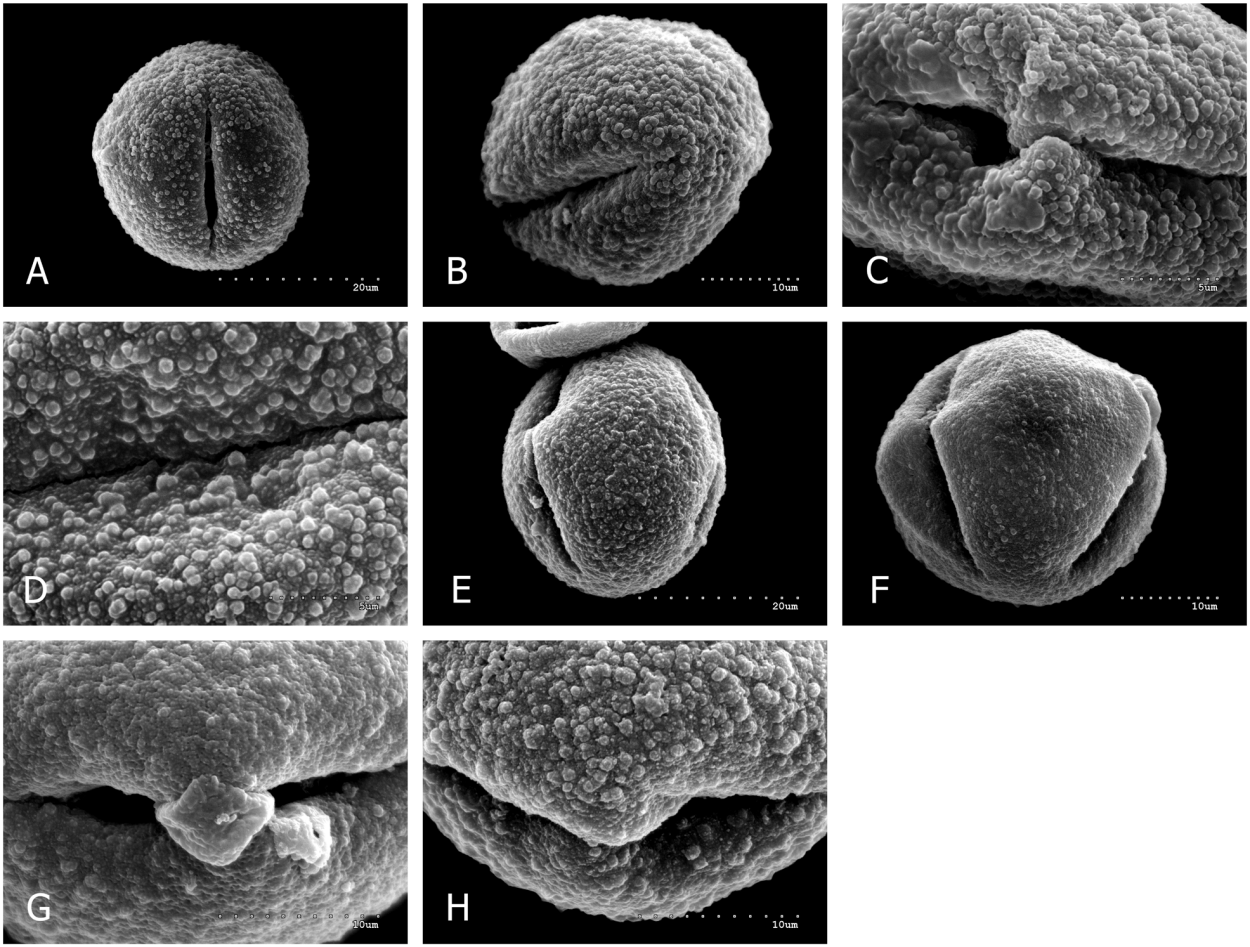
*Q*. *petraea* × *Q*. *pubescens*, A-D. A, equatorial view; B, polar view with one ectocolpus; C, ectocolpus and exine granulate-verrucate ornamentation; D, exine ornamentation details—see (Fig 1E); *Q*. *robur × Q*. *petraea*, E-H. E, equatorial view, F, polar view with two ectocolpi; G, ectocolpus and exine granulate-verrucate ornamentation; H, exine ornamentation details—see (Fig 1E).

Quantitative features of pollen grains are summarized in Table 2 and illustrated with scanning electron micrographs (Figs 1A–1M and 2A–2H). Pollen grains of the examined taxa are monads, isopolar, radially symmetrical. The pollen grains are tricolporoidate or tricolpate.

**Table 2:** Range (min-max), mean values and coefficient of variation (cv) of studied features. One-way ANOVA’s were performed separately for each of traits. Same letters indicate a lack of statistically significant differences between analyzed taxa according to Tukey’s *post hoc* test (p < 0.001).

**Feature**	**P**			**E**			**P/E**		
**Species**	Min-Max	Mean	CV (%)	Min-Max	Mean	CV (%)	Min-Max	Mean	CV (%)
*Q*. *petraea*	22–40	31.37 b	8.01	20–40	30.26 bc	9.60	0.8125–1.5	1.043 b	9.92
*Q*. *pubescens*	24–42	31.78 b	8.69	22–38	30.31 bc	7.94	0.8–1.545	1.052 b	9.62
*Q*. *robur*	24–42	30.79 b	9.32	20–38	29.26 c	9.69	0.75–1.636	1.060 b	11.56
*Q*. *petraea × Q*. *pubescens*	28–38	32.00 b	5.85	24–36	31.24 ab	7.65	0.8333–1.417	1.030 b	9.34
*Q*. *robur × Q*. *petraea*	32–42	37.07 a	7.05	30–40	32.80 a	6.52	1–1.312	1.133 a	7.71
LSD_0.001_		1,63			1,69			0.067	
*F* statistic		46.04***			27.94***			7.19***	
**Feature**	**Exp**			**Exe**			**Exp/P**		
**Species**	Min-Max	Mean	CV (%)	Min-Max	Mean	CV (%)	Min-Max	Mean	CV (%)
*Q*. *petraea*	0.4–2	1.025 bc	41.96	0.4–2	1.017 bc	40.68	0.0111–0.0909	0.0329 a	43.17
*Q*. *pubescens*	0.6–2	1.066 abc	37.94	0.6–2	1.033 bc	35.61	0.0158–0.0769	0.0337 a	38.39
*Q*. *robur*	0.6–2	1.182 ab	38.45	0.6–2	1.150 ab	37.66	0.0158–0.0769	0.0387 a	39.33
*Q*. *petraea × Q*. *pubescens*	0.6–2	1.304 a	37.37	0.6–2	1.291 a	37.17	0.0167–0.0714	0.0408 a	37.39
*Q*. *robur × Q*. *petraea*	0.6–1.4	0.880 c	27.09	0.6–1.4	0.853 c	26.07	0.0150–0.0368	0.0237 b	25.36
LSD_0.001_		0.266			0.253			0.009	
*F* statistic		19.59***			19.02***			24.89***	
**Features**	**Exe/E**			**Le**			**Le/P**		
**Species**	Min-Max	Mean	CV (%)	Min-Max	Mean	CV (%)	Min-Max	Mean	CV (%)
*Q*. *petraea*	0.012–0.09091	0.03402 ab	43.12	16–36	26.18 b	11.92	0.5714–1	0.835 b	8.83
*Q*. *pubescens*	0.01579–0.07692	0.03427 ab	36.39	18–38	26.97 b	11.62	0.4737–1	0.849 ab	7.62
*Q*. *robur*	0.0117–0.1	0.0399 a	40.43	18–38	26.32 b	13.04	0.6000–1	0.854 ab	8.25
*Q*. *petraea × Q*. *pubescens*	0.01765–0.08333	0.04194 a	40.82	20–32	27.31 b	9.89	0.6875–1	0.853 ab	7.87
*Q*. *robur × Q*. *petraea*	0.015–0.04375	0.02620 b	27.86	28–36	32.07 a	6.85	0.7778–1	0.867 a	5.85
LSD_0.001_		0.009			1.964			0.023	
*F* statistic		23.44***			28.66***			8.89***	

P—length of polar axis; E—equatorial diameter; Le—length of ectocolpi; Exp—thickness of exine along polar axis; Exe—thickness of exine along equatorial diameter.

*** P<0.001.

According to Erdtman’s [80] pollen size classification, the studied pollen grains of parental species were medium—99% (25.1–50 `m), very rarely (1%) small-sized (10.0–25.0 μm). The size of all pollen of hybrids was medium (Table 2).

Parental species exhibited smaller pollen grains as compared to hybrids. The average length of the polar axis (P) in parental *Quercus* species was 31.24 (22.00–42.00) μm and in hybrids—33.27 (28.00–42.00) μm. In parental species, the shortest polar axis (P) occurred in the pollen of *Q*. *petraea* (22.00 μm), while the longest one—in *Q*. *robur* and *Q*. *pubescens* (42.00 μm). For hybrids, the shortest polar axis (P) was found in *Q*. *petraea × Q*. *pubescens* (28.00 μm) and the longest one—in *Q*. *robur × Q*. *petraea* (42.00 μm; Table 2). The mean length of the equatorial diameter (E) in parental *Quercus* species amounted to 29.90 (20.00–40.00) μm and in hybrids—31.63 (24.00–40.00) μm.

In all studied taxa, the outline in polar view was mostly circular, more rarely triangular or elliptic, whereas in equatorial view it was mostly elliptic or circular.

The mean P/E ratio in parental *Quercus* species was 1.05 and ranged from 0.75 to 1.64 in *Q*. *robur* and in hybrids it was 1.06 (range 0.83–1.42) in *Q*. *petraea × Q*. *pubescens* (Table 2). With respect to features, pollen shapes of parental species and hybrids were different (Table 3). In the case of parental species, most frequent pollen grains were prolate-spheroidal (36.2%), spheroidal (26.6%) and oblate-spheroidal (20.4%), while subprolate ones occurred more rarely (14.2%), prolate (1.5%) and suboblate (1.1%) pollen were found only sporadically. In hybrids, spheroidal (32.5%) and prolate-spheroidal (30.8%) pollen grains were most common, while oblate-spheroidal and subprolate (17.5% each) were not so frequent and prolate and suboblate pollen were encountered only in single grains (0.8% each). Slightly different results were obtained when analysing the distribution of pollen shape class in individual taxa. In the case of parental species, the results were similar to those reported above, but in hybrids—they differed significantly both from one another and from parental species (Table 3). *Quercus robur × Q*. *petraea* was distinguished by the highest number of elongated pollen grains (subprolate—40% and prolate-spheroidal—46.7%). In *Q*. *robur × Q*. *petraea*—spheroidal pollen were not numerous (13.3%), while oblate-spheroidal pollen—fairly frequent in other taxa (17.2–24.4%)—did not occur at all. On the other hand, *Q*. *petraea × Q*. *pubescens*, in contrast to *Q*. *×rosacea*, exhibited most frequently spheroidal (38.9%) and oblate-spheroidal (24.4%) pollen accompanied by the lowest proportion of prolate-spheroidal pollen (25.6%) among the studied taxa.

**Table 3:** The percentage participation of pollen grains in shape classes (P/E ratio) according to Erdtman’s (1952) classification.

Taxon	Pollen shape classes					
	suboblate	oblate-spheroidal	spheroidal	prolate-spheroidal	subprolate	prolate
*Q*. *petraea*	1.2	20.9	27.8	36.9	12.1	1.1
*Q*. *pubescens*	0.8	16.4	29.0	42.1	10.5	1.3
*Q*. *robur*	1.2	22.0	23.8	32.0	18.8	2.2
*Q*. *petraea × Q*. *pubescens*	-	24.44	38.89	25.56	10.00	1.11
*Q*. *robur × Q*. *petraea*	-	-	13.33	46.67	40.00	-

Suboblate (0.75–0.88); oblate-spheroidal (0.89–0.99); spheroidal (1.00); prolate-spheroidal (1.01–1.14); subprolate (1.15–1.33); prolate (1.34–2.00).

The mean exine thickness was 1.08 (0.4–2.0) μm (parental species) and 1.19 (0.60–2.0) μm (hybrids; Table 2). Exine was thinnest in *Q*. *petraea* (0.4 μm) and thickest (2.0 μm) among all studied species. In hybrids, exine thickness varied the least, from 0.6 μm in both studied taxa to 2.0 μm—in *Q*. *petraea × Q*. *pubescens*. The relative thickness of the exine (Exp/P ratio) was similar for parental species and hybrids amounting to 0.04 (0.01–0.1) and 0.04 (0.02–0.08), respectively.

Pollen grains had three apertures and were tricolpate or tricolporoidate (this is in angiosperm pollen a rare character, when ectoaperture consists of a colpus with an indistinct endoaperture). Colpi were arranged meridionally, regularly. They were very narrow, with acute to narrowly obtuse ends. Commonly colpi were covered at the equator by a geniculum—a bulge formed by sexine extensions. Colpus membranes were usually smooth. Colpi were long; mean length in parental species—26.40 (16–38) μm and in hybrids—28.50 (20–36) μm (Table 2). On average, the length of colpi in parental species comprised 84% of the polar axis length and in hybrids—86%. Therefore, parental species, on average, exhibited slightly shorter colpi in comparison with hybrids. Their width was variable and usually greatest in the equatorial region. An endoaperture was absent to clearly-developed.

In all studied taxa, exine ornamentations in SEM were granulate or granulate-verrucate, because they were made up, primarily of granules less than 1μm in size (usually measuring from 0.5 to 1μm), and less frequently greater than 1μm verrucae (wart-like elements, broader than high; Hesse et al. [72]) (Figs 1E, 1H, 1M, 2D and 2H). In all the examined taxa, with the exception of *Q*. *pubescens* (Fig 1H), small and low granules, usually measuring from 0.1 to 0.3 μm, also occurred profusely. Perforations of varying diameters were minor, scarce to numerous, and sometimes not observed.

**Fig 3:**
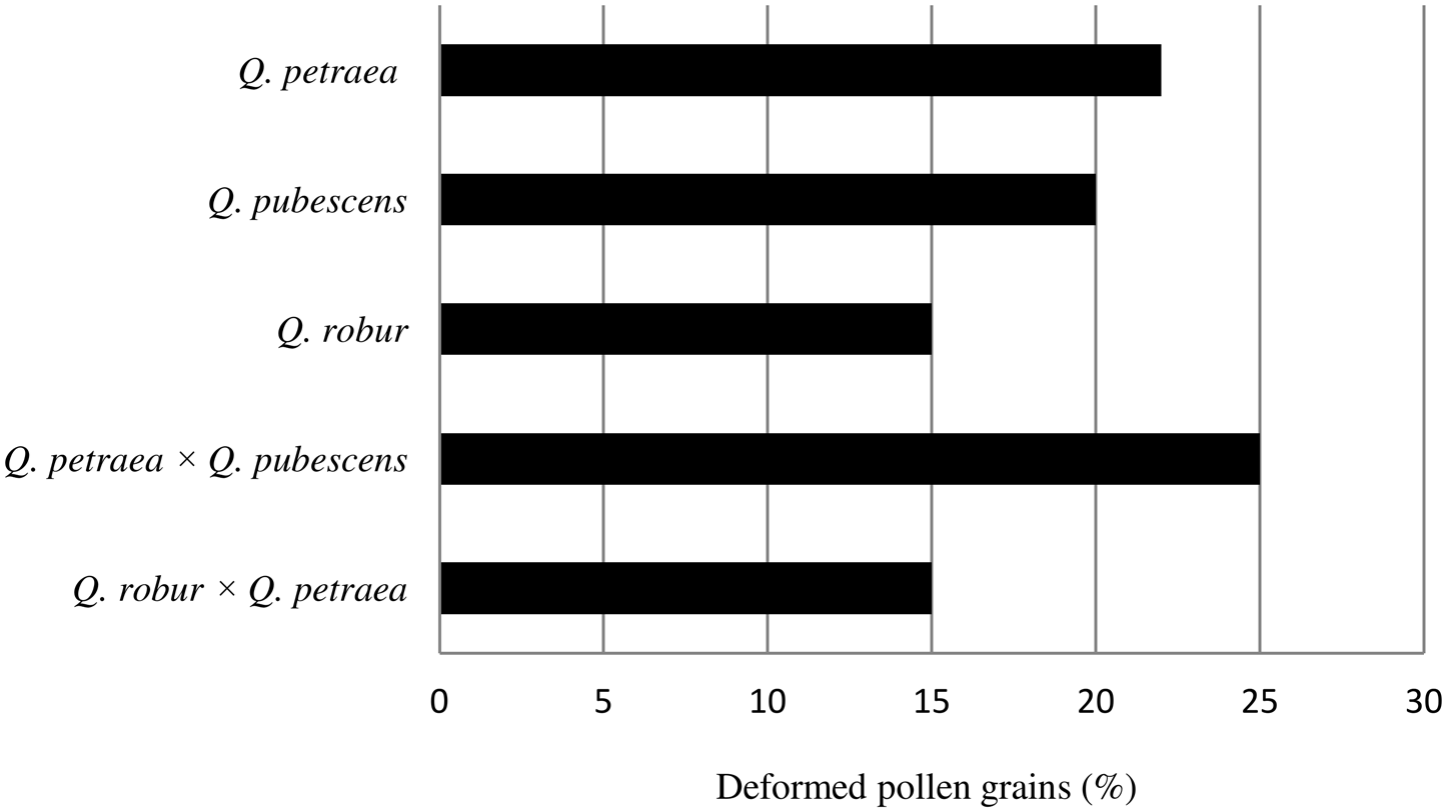
Percentage of deformed pollen grains.

On average, the percentage share of deformed pollen grains in the samples (from 200 to 1000 grains per taxon) was similar and ranged from 15% in *Q*. *robur* and *Q*. *robur × Q*. *petraea* to 25% in *Q*. *petraea × Q*. *pubescens* (Fig 3). The highest frequency of deformed pollen was found in samples of *Q*. *petraea* and *Q*. *petraea × Q*. *pubescens* (30%) and the lowest in *Q*. *robur* (10%). In parental species, the lowest percentage of deformed pollen grains was observed in samples of *Q*. *robur* (10%); 20% in *Q*. *pubescens* and 30% in *Q*. *petraea*. On average, deformed pollen occurred at frequencies of 15, 20 and 22%, respectively, in the three species. In hybrids, the percentages of deformed pollen grains were: 15% in *Q*. *robur × Q*. *petraea* and 25% in *Q*. *petraea × Q*. *pubescens*. Many well-preserved pollen grains were found in the majority of the samples. The deformations consisted mainly in local ruptures of pollen grains, nearly always in the aperture area, and their slight flattening due to reduced turgor. A small number of pollen grains were burst in the area of apertures and strongly deformed to the extent that they had unusual shapes and outlines caused by almost complete loss of turgor and strong flattening. Our observations in LM and SEM were made on acetolysed grains. The experience of the authors of the article allows for a statement that pollen prepared in such manner are subject to deformation in the process of acetolysis, under the influence of high temperature or impact of concentrated acids, as well as in the course of coating with gold target prior to observations in SEM, and in vaccum in SEM, when stream of electrons falls upon them. Due to such actions pollen burst and in consequence lose turgor.

### Interspecific variability of pollen grains

**Fig 4:**
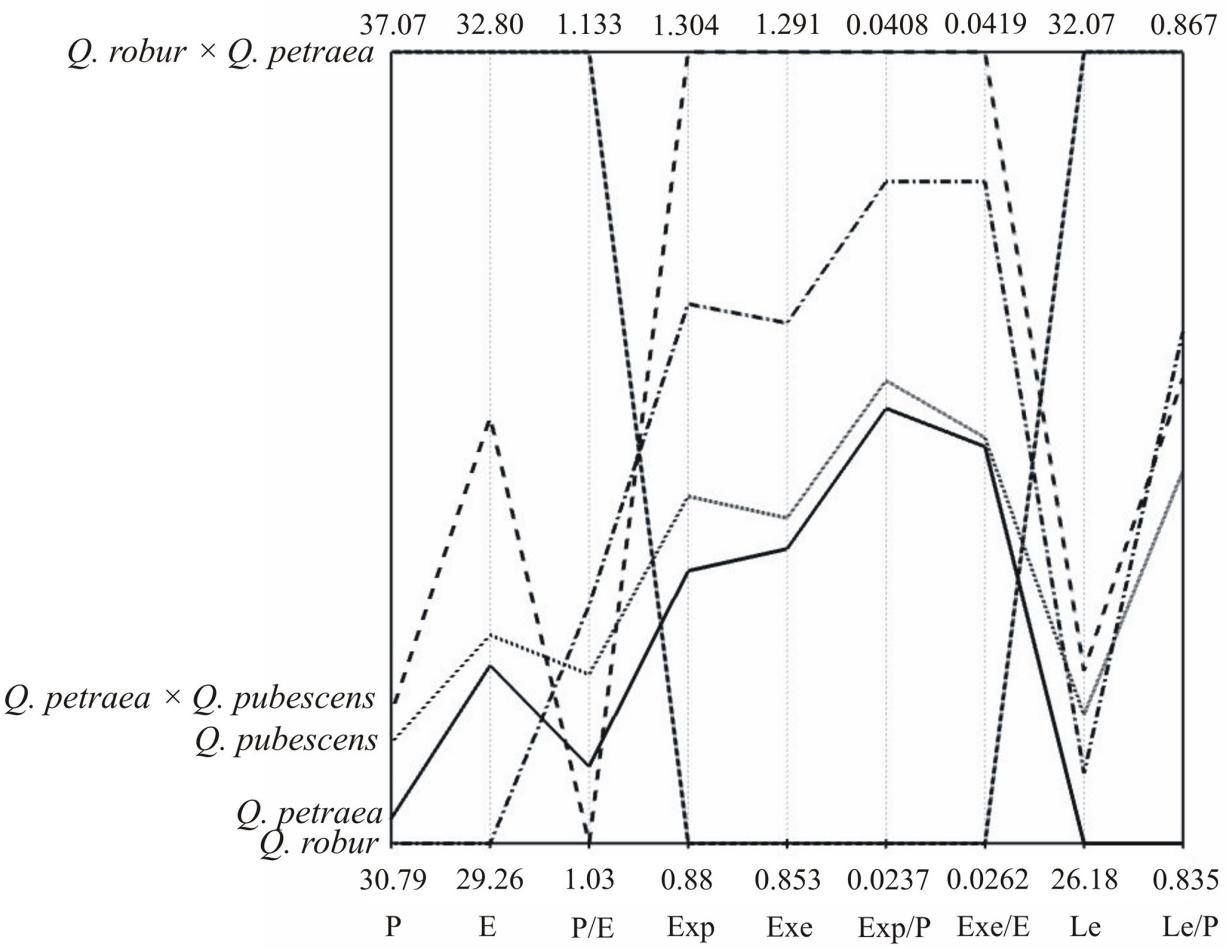
Parallel coordinate plots (PCPs) for five studied taxa and nine traits (P, E, P/E, Exp, Exp/P, Le, Le/P).

Results of the performed MANOVA indicate, that all taxa were significantly (Wilk’s λ = 0.7984; F_36,7482_ = 12.87; *P* < 0.0001) different for all nine traits. The analysis of variance for nine biometric traits [P (F_4,2004_ = 46.04), E (F_4,2004_ = 27.94), P/E (F_4,2004_ = 7.19), Exp (F_4,2004_ = 19.59), Exe (F_4,2004_ = 19.02), Exp/P (F_4,2004_ = 24.89), Exe/E (F_4,2004_ = 23.44), Le (F_4,2004_ = 28.66) and Le/P (F_4,2004_ = 8.89)] confirmed the variability of the tested taxa at the significance level α = 0.001 (Table 2). Mean values and coefficients of variations (CV) for the observed traits indicate high variability among the tested taxa for which significant differences were found in terms of all analysed morphological traits (Table 2, Fig 4).

The performed correlation analysis indicates statistically significant correlation coefficients of 29 out of 36 coefficients (Table 4). In the case of seven pairs of traits, no significant correlation was established of: Exp with P, Exp/P with P/E, Le with Exp, Le with Exe, Le/P with Exp, Le/P with Exe and Le/P with Exp/P. Seventeen out of 29 significantly correlated pairs of traits were characterised by positive correlation coefficients. This means that a value increase of one trait in a given pair leads to a value increase of the second trait.

**Table 4:** The correlation matrix for the observed features.

Feature	P	E	P/E	Exp	Exe	Exp/P	Exe/E	Le	Le/P
P	1								
E	0.394***	1							
P/E	0.492***	-0.598***	1						
Exp	0.006	-0.111***	0.110***	1					
Exe	-0.045*	-0.099***	0.055*	0.851***	1				
Exp/P	-0.216***	-0.192***	-0.007	0.971***	0.842***	1			
Exe/E	-0.133***	-0.331***	0.201***	0.828***	0.966***	0.843***	1		
Le	0.742***	0.349***	0.308***	-0.010	-0.038	-0.178***	-0.122***	1	
Le/P	0.045*	0.101***	-0.060**	-0.026	-0.014	-0.041	-0.045*	0.701***	1

See explanations to Table 2.

* P<0.05;

**P<0.01;

***P<0.001.

The greatest differentiation of all the analysed phenotypic traits expressed jointly with the greatest Mahalanobis distance was recorded for the pollen grain of *Q*. *robur × Q*. *petraea* (Table 5). Pollen grains of *Q*. *robur × Q*. *petraea* differed significantly with respect to all the examined traits from the remaining taxa. In turn, the greatest phenotypic similarity was observed for *Q*. *robur* and *Q*. *petraea × Q*. *pubescens* (0.915), *Q*. *petraea × Q*. *pubescens* and *Q*. *petraea* (0.881) as well as for *Q*. *petraea × Q*. *pubescens* and *Q*. *pubescens* (0.821).

**Table 5:** Phenotypic distance between the taxa calculated on the basis P, E, P/E, Exp, Exp/P, Le, Le/P by Mahalanobis distance.

Taxon	*Q*. *petraea*	*Q*. *pubescens*	*Q*. *robur*	*Q*. *petraea × Q*. *pubescens*	*Q*. *robur × Q*. *petraea*
*Q*. *petraea*	0				
*Q*. *pubescens*	0.344	0			
*Q*. *robur*	0.881	0.821	0		
*Q*. *petraea × Q*. *pubescens*	0.619	0.599	0.915	0	
*Q*. *robur × Q*. *petraea*	2.931*	2.782*	3.089**	3.122**	0

* P<0.05;

** P<0.01.

**Fig 5:**
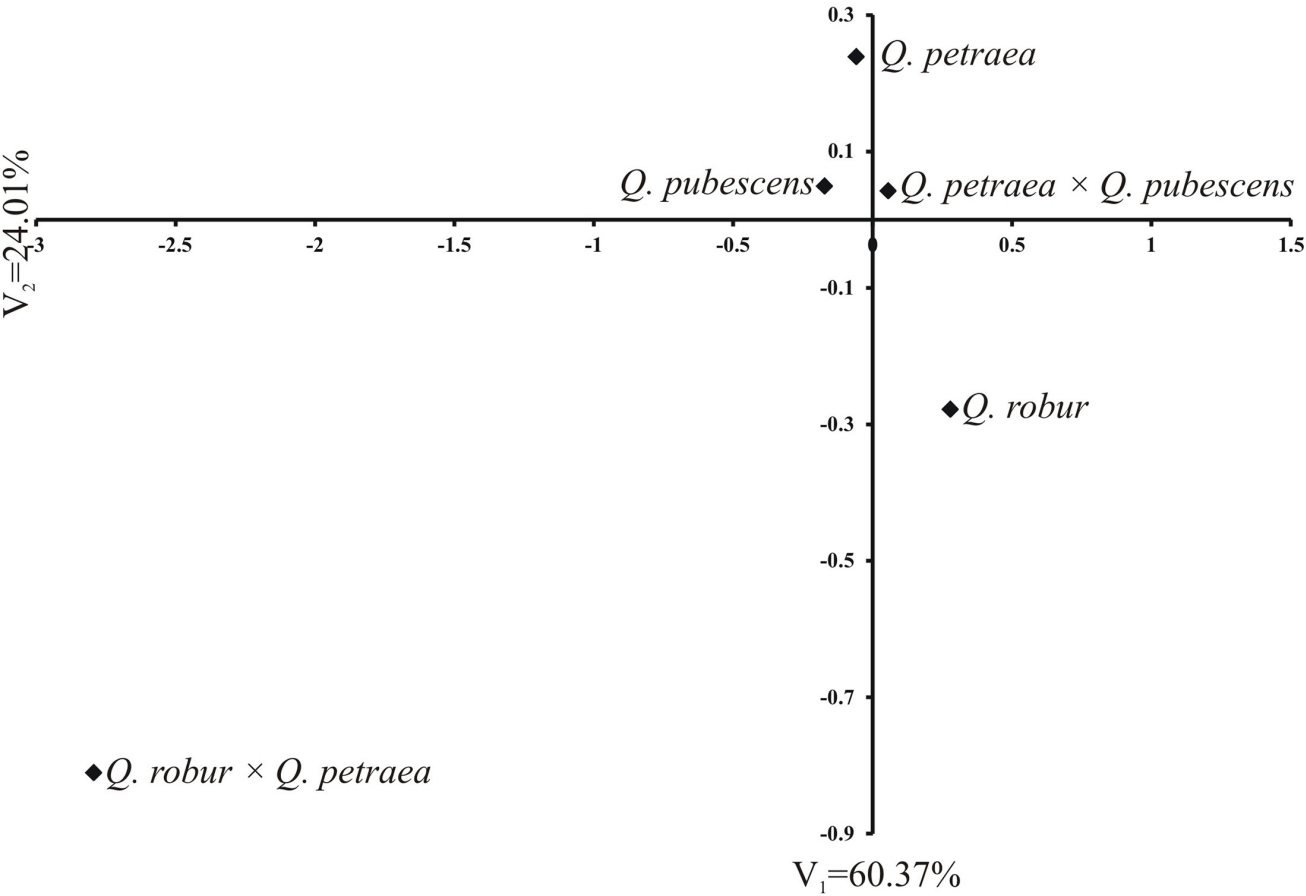
Distribution of five *Quercus* taxa studied in the space of two first canonical variables.

The first two canonical variables accounted for 84.38% of total multivariate variability between species and hybrids (Fig 5). This diagram of the first two canonical variables was used to divide the studied taxa into three groups. The first group comprised *Q*. *petraea*, *Q*. *pubescens* and *Q*. *petraea* × *Q*. *pubescens*, the second one included one taxon—*Q*. *robur* and the last group also embraced just one taxon *Q*. *×rosacea*, which was very distant from the remaining groups (Fig 5).

Interesting results were obtained by the contrast analysis between parental species and their hybrids (Table 6). With respect to P, Le and E features, and to a lesser degree, also P/E, pollen grains of *Q*. *robur × Q*. *petraea* exhibited significantly and considerably higher means in comparison with the mean value of its parental forms (negative value of the contrast). In the case of Exp, Exe, Exp/P, Exp/E traits, mean values for *Q*. *robur × Q*. *petraea* were statistically significantly smaller, than the mean value of *Q*. *robur* and *Q*. *petraea*. Only for Le/P, there were no statistically significant differences between the mean values for hybrids and the parental forms (Table 6). The *Q*. *pubescens × Q*. *petraea* hybrid was characterised by statistically significantly higher mean values of P, E, Exp, Exe, Exp/E and Exe/E traits than its parental forms. Only for P/E, the *Q*. *petraea × Q*. *pubescens* hybrid outlined a lower mean from parental species (positive contrast value).

**Table 6:** Results of contrasts analysis between parental species and their hybrids.

Feature	Contrast	
	*Q*. *robur*, *Q*. *petraea* and *Q*. *robur × Q*. *petraea*	*Q*. *petraea*, *Q*. *pubescens* and *Q*. *petraea × Q*. *pubescens*
P	-5.97 ***	-0.61 *
E	-3.00 ***	-1.38 ***
P/E	-0.082 ***	0.027 *
Exp	0.217 **	-0.187 ***
Exe	0.225 **	-0.204 ***
Exp/P	0.0119 ***	-0.0050 **
Exe/E	0.0105 ***	-0.0051 **
Le	-5.82 **	-0.57
Le/P	-0.023	-0.0016

See explanations to Table 2.

* P<0.05;

** P<0.01;

*** P<0.001.

**Fig 6:**
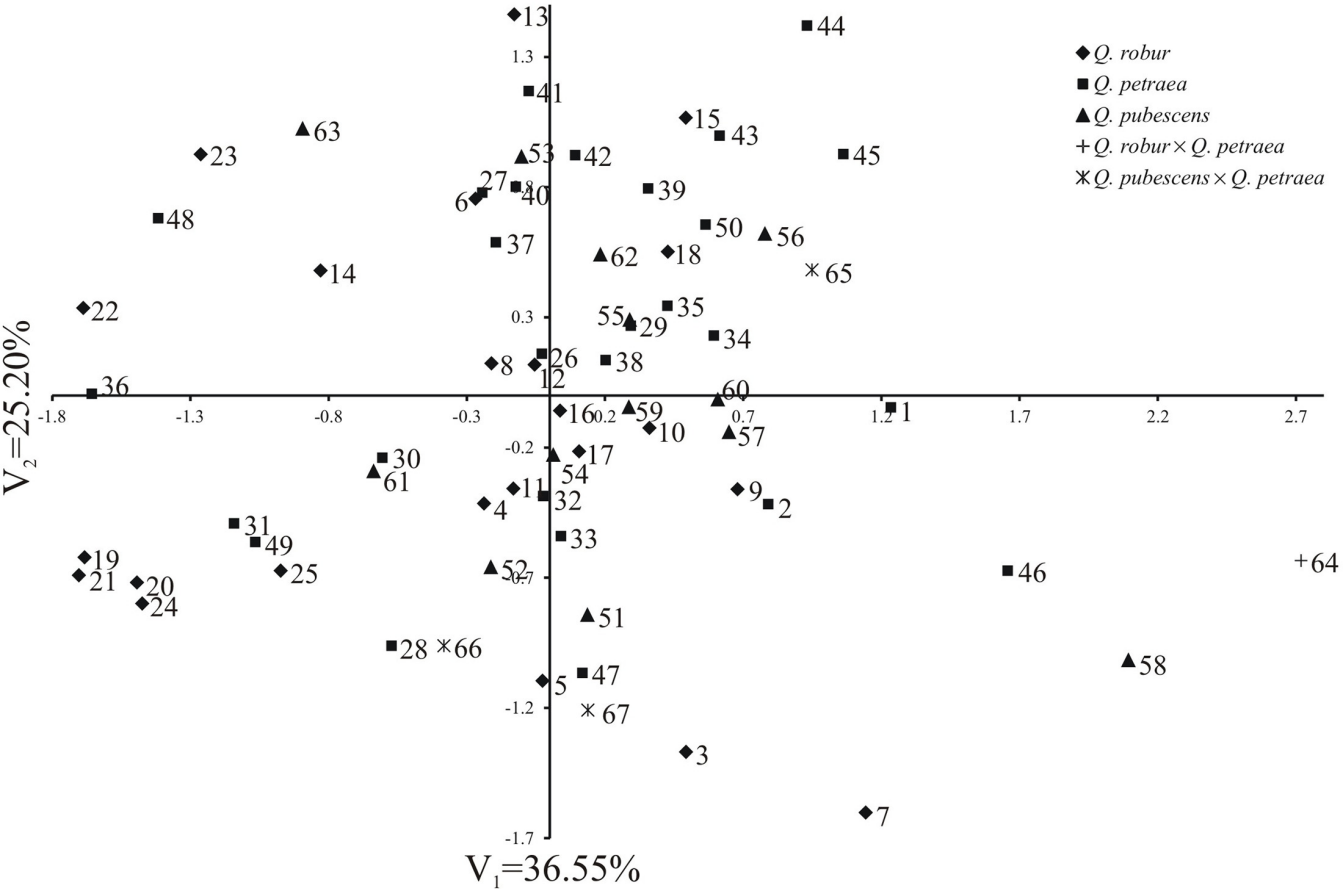
Distribution of 67 *Quercus* trees studied in the space of two first canonical variables.

Fig 6 shows the variability of pollen grain traits of 67 studied *Quercus* individuals in the configuration of the first two canonical variables. On the graph, the coordinates of the point for particular trees are values of the first and second canonical variables, respectively. The first two canonical variables accounted for 61.75% of the total multivariate variability between individual trees. The goal of the study was to establish whether pollen grains collected from various oak trees growing in different habitat conditions (soil, climate) would differ from one another. Six groups of trees were distinguished (Fig 6). The majority of the examined individuals was found in the first group (I). To the other five groups (II-VI) belongs a few trees (II—14, 22, 23 36, 48 and 63, III—19–21, 24, 25, 30, 31, 49, 61, IV—44, 45, V—7, 46, 58, VI—64 (Fig 6). The analysis of the sites, from which flowers (pollen grains) from individual oak trees were collected, has shown, that in individual groups, both trees derived from the same sites [e.g. in group I, occur all analysed *Q*. *petraea* trees from Rokita (41–43) or nearly all *Q*. *pubescens* trees from Bielinek (51–57, 59–60)] as well as from places geographically distant from one another [e.g. from Austria—*Q*. *pubescens* (62) or from Poland—*Q*. *robur* from distant Białowieża and Bukowa Primeval Forest. A similar situation occurred also in smaller groups, for example, in group V—each of the three trees represents a different species derived from a different place (*Q*. *robur* from Wielkopolski National Park—7, *Q*. *petraea* from Bukowa Primeval Forest—46, *Q*. *pubescens* from Bielinek—58). Only group IV is made up of two oaks derived from the same place—Bukowa Primeval Forest.

## Discussion

Palynological investigations on pollen grain features of parental species and their interspecific hybrids focus on comparing pollen size and much rarely involve proportions of deformed pollen grains in both these groups. According to some palynologists, hybrids have significantly larger pollen grains than those of their parents [58, 64, 81–83]. Also *Quercus* taxa investigated in the present study belong to this group because—as in the case of mean values of P and E features (pollen size), as well as for individual taxa—hybrids had greater pollen grains than parental species. Other researchers proved that hybrids can have pollen size similar or smaller than their parents [53, 55, 57, 60, 62, 66, 84, 85]. Last but not the least, there are also cases where some hybrids are characterised by pollen grains larger than parental species, while others—smaller [59, 61, 64].

Among the studied parental *Quercus* species, it was found that *Q*. *petraea* and *Q*. *pubescens* exhibited pollen grains most similar to each other. *Q*. *robur* differed from them on average, smaller pollen size and greater exine thickness (Table 2, Fig 5). In hybrids, the dissimilarity of *Q*. *robur × Q*. *petraea* pollen grain features was more conspicuous than in all the remaining taxa. It is worth emphasising, that the oak from which the pollen grains derived, exhibited quite distinct hybrid morphological features. On the basis of contrast analysis, this taxon had the largest pollen grains of longest colpi, significantly bigger with respect to P, E, P/E and Le traits than the mean value of its parental forms. At the same time, it exhibited a fairly thin exine; therefore, mean values of traits associated with it (Exp, Exe, Exp/P, Exp/E) for *Q*. *robur × Q*. *petraea* were smaller in comparison with *Q*. *robur* and *Q*. *petraea*. Hybrid *Q*. *petraea × Q*. *pubescens*, even though, did not distinguish itself so clearly as *Q*. *robur* and *Q*. *petraea* (Fig 5). It was also characterised by larger mean values of nearly all analysed traits than in parental forms, including exine features (P, E, Exp, Exe, Exp/E and Exe/E) (Table 6). The hybrids derived from Bielinek on the Oder (NW Poland) to hybrids between *Q*. *petraea* and *Q*. *pubescens*. The phenomenon of crossing of *Q*. *pubescens* mainly with *Q*. *petraea* in a peculiar, strongly isolated as well as most distant population of this species from its dense range in Bielinek on the Oder was stressed by Staszkiewicz [86], Danielewicz et al. [87] as well as Krzakowa et al. [88]. However, in recent years, on the basis of genetic analyses employing 14 nuclear microsatellites as markers, it was found that degree of relationship between *Q*. *pubescens* individuals was considerable. It implies that crossing in the population occurs, to a large extent, between related individuals and, to a lesser degree, with other species [89]. This, by no means, indicates that interspecific hybrids do not occur there at all, but shows their smaller frequency.

Despite numerous palynological studies, descriptions of several important morphological features of *Quercus* pollen grains are not clear. This refers, in particular, to a very diverse, among representatives of this genus, exine ornamentation but also to endoaperture types. Also, data regarding perforation numbers are not accurate [38, 43, 62]. Palynologists give different types of exine ornamentation in SEM in different representatives of the *Quercus* genus. It can be either micro-rugulate, scabrate or scabrate-verrucate with verrucae beset with small, rounded processes [36], scabrate, microscabrate or microverrucate-scabrate [90], granulate, scabrate and microgranulate [62], granulate [72], verrucate or microverrucate [38], psilate-verrucate, verrucate, scabrate, scabrate-verrucate and psilate-scabrate [43] or microverrucate to verrucate, rarely regulate-granulate [90]. The three study species were either granulate or granulate-verrucate. This type of exine ornamentation has been selected because it is made primarily, of granules which are less than 1 μm (usually—05–1 μm), whereas verrucae exceed 1 μm [72].

In all studied taxa, with the exception of *Q*. *pubescens*, besides larger granules and verrucae also numerous smaller granules, commonly measuring 0.1–0.3 μm, occurred. Dissimilarity of *Q*. *pubescens* exine ornamentation compared to all studied *Quercus* taxa is also corroborated by Benthem et al. [36], Colombo et al. [17] and Smit [44]. The results of the present study agree with Benthem et al. [36] as well as Hesse et al. [72] that colpate or colporoidate pollen grains occurred in the investigated *Quercus* taxa. The latter ones were composed of a colpus (ectoaperture) with an indistinct endoaperture. Some researchers maintain, that both poroides as well as pori [43], or only pori [17, 44, 91–92] occur here, while others—in some part of the species (e.g. *Q*. *pubescens* Willd., *Q*. *aristata* Hook. & Arn., *Q*. *dumosa* Nutt., *Q*. *laurina* Bonpl.)—also find absence of the endoapertures [17, 47, 48].

The number of perforations differs, depending on authors; some report their total absence or only a few and others mention many with differing diameters and distributions on pollen grains [17, 36, 43, 47, 48, 91]. Results of this study corroborate the above observations; perforations were small, scarce or numerous, sometimes they could not be seen at all. They had different diameters and were usually distributed irregularly.

The results of statistical analyses are not unequivocal both with respect to the share of the 67 individuals (oak trees) in 6 groups to which they were assigned as well as to places of their collection. The majority of the investigated individuals belonged to the first, large group, while the remaining ones occurred from single to several oak trees in the other five groups. In these groups, both individuals from different taxa (e.g. group 5 is made up of three individuals—*Q*. *petraea*, *Q*. *pubescens* and *Q*. *robur*) as well as trees representing the same taxa (e.g. group 4—*Q*. *petraea*) were found. Flowers from five *Q*. *pubescens* trees with traits typical for this species (51–55) were collected from the site in Bielinek Reserve (Poland) and, for comparison, from five other *Q*. *pubescens* trees (56–60) derived from Bielinek and growing in the Dendrological Garden of Poznań University of Life Sciences. Pollen grains of all these trees were similar to one another, because almost all of them were found in the same group—group 1 and only one tree (58) belonged to group 5. A similar situation was observed with geographical distribution. In the same group occurred both trees growing in the same site (e.g. in group 1—three *Q*. *petraea* trees from Rokita) as well as oaks derived from geographically distant sites (in group 2—*Q*. *robur* from Spain and from Dębno in Poland).

Not much information can be found in the literature on the subject concerning deformed pollen grains. Some palynologists maintain that the share of deformed pollen grains is greater in hybrids, than in parental species. Karlsdóttir et al. [60] reported that the in natural birch hybrids it was found two to three times more abnormal pollen than in parental species *Betula nana* and *B*. *pubescens*. However, in three parental *Crataegus* species and in their three natural hybrids, deformed pollen grains occurred with similar frequencies (20–40%) [64]. The results on *Quercus* pollen grains reported here are similar. The proportions of deformed pollen in parental *Quercus* species and hybrids were similar and, on average, represented by ap to 15–25%.

Recapitulating, it was to be expected that not all of the closely related species of oaks can be safely distinguished using pollen morphology. In spite of such close relationships of the examined *Quercus* taxa, it was, nevertheless, possible to identify two (*Q*. *robur × Q*. *petraea* and *Q*. *pubescens*) from among five taxa on the basis of several analysed pollen features. Pollen size can be used as an auxiliary feature when diagnosing *Quercus* parental species and hybrids. Pollen shape is an interesting, hitherto omitted trait, which distinguishes both hybrids, especially *Q*. *robur × Q*. *petraea* characterised by the most elongated pollen grains. On the basis of exine ornamentation, it was possible to identify only *Q*. *pubescens*; the remaining species as well as hybrids did not differ significantly with regard to this feature. Only a greater number of such studies, based on large pollen samples, will show if there really is signal in pollen shape or exine ornamentation to tell species and hybrids apart. The determination of the diagnostic value of endoapreture features, i.e. their type (pori, poroides or both of these aperture types) as well as their presence or absence requires further comprehensive palynological investigations.
